# A preliminary study investigating the neglected domain of mental health in Australian lifesavers and lifeguards

**DOI:** 10.1186/s12889-023-15741-5

**Published:** 2023-05-31

**Authors:** Samantha Fien, Jasmin C. Lawes, Jessica Ledger, Murray Drummond, Pamela Simon, Nancy Joseph, Shane Daw, Talitha Best, Robert Stanton, Ian de Terte

**Affiliations:** 1 School of Health, Medical and Applied Sciences, Central Queensland University, Mackay, QLD Australia; 2 Research Cluster for Resilience and Wellbeing, Appleton Institute, Wayville, South Australia; 3 Surf Life Saving Australia, Bondi Beach, Sydney, NSW Australia; 4 College of Education, Psychology and Social Work, SHAPE Research Centre, Flinders University, Adelaide, Health Australia; 5 School of Health, Medical and Applied Sciences, NeuroHealth Lab, Appleton Institute, Central Queensland University, Brisbane, QLD Australia; 6 School of Health, Medical and Applied Sciences, Central Queensland University, Rockhampton, QLD Australia; 7 School of Psychology, Massey University, Wellington, New Zealand; 8 School of Health, Medical and Applied Sciences, Central Queensland University, Building 4, Room G.33, Mackay City Campus, Sydney Street, Mackay, Mackay, QLD 4740 P +61 7, 4940 3430E Australia

**Keywords:** Mental health, Lifeguards, Lifesavers, First responders, Post-Traumatic Stress Symptoms (PTSS), Volunteers, Australia

## Abstract

**Background:**

Surf lifesavers and lifeguards have provided essential education, preventative, and rescue services to the Australian community for over 110 years. In this first responder role, surf lifesavers and lifeguards are inadvertently exposed to high risk and trauma related experiences, which may negatively impact mental well-being. To date however, there has been limited research into the mental health of surf lifesavers and lifeguards, and no studies at all on the mental health of adolescent surf lifesavers. The preliminary study aimed to measure the exposure of potentially traumatic events (PTEs), post-traumatic stress symptoms (PTSS), self-efficacy, social support, and attitudes towards mental health problems in Surf Life Saving (SLS) members.

**Methods:**

An anonymous, online survey was developed (adolescent and adult versions) and created to measure the domain of mental health in surf lifesavers and lifeguards. Pearson’s correlations investigated relationships between PTEs, PTSS, self-efficacy, social support, attitudes towards mental health problems, age, years as a SLS member, and years patrolling. Spearman’s Rank was used for violations of normality.

**Results:**

A total of 57 surf lifesavers/lifeguards aged 13–59 years were included in the final analysis. There was a significant positive relationship between exposure to direct trauma and PTSS, which in turn, were associated with greater negative attitudes towards mental health problems towards the mental health of others, and lower levels of self-efficacy. Male and female adults with PTSS reported lower social support, whereas for adolescent males, a positive relationship between direct trauma and PTSS was observed.

**Conclusion:**

This research is the first to explore the mental health of Australian surf lifesavers and lifeguards. The results highlight the potential risks to mental health and well-being associated with this first responder role. More research to protect the vulnerability of this population is warranted.

**Supplementary Information:**

The online version contains supplementary material available at 10.1186/s12889-023-15741-5.

Clinical impact statement: This research is the first to explore the mental health of Australian surf lifesavers and lifeguards and the first globally to address adolescent first responders. The significant relationships found in the adult participants were in the scoring of a higher level of self-efficacy and social support revealing a lower level of PTSS. Higher levels of PTSS were also related to negative attitudes towards mental health problems in the adult participants. Meanwhile, adolescents found significant relationships between PTEs (global and within SLS) and PTSS for adolescents, as well as in the scoring of higher social support scores relating to an increase in PTSS.

## Introduction

The Surf Life Saving movement comprises volunteer surf lifesavers and paid lifeguards, with the majority being volunteer surf lifesavers. This role provides essential emergency services and assistance to people who are injured or may be at risk on the coast. This can place Surf Life Saving members (herein SLS) at personal risk of exposure to potentially traumatic events (PTEs) such as drowning deaths, shark attacks, natural hazards, and suicides [1–3]. Our understanding of the impact of such incidents on SLS is limited to a single study from New Zealand [4], although recent reports have identified significant mental health concerns in other first responder populations [5, 6]. Overall, emergency service personnel experience higher psychological distress and mental ill health compared to the general population [5, 6]. Despite being investigated in other first responders and emergency service personnel, such as, police officers [7], military personnel [8], fire fighters [9], and paramedics [10], the potential impacts of continued exposure to PTEs in SLS has only recently been highlighted [11] and remains unknown.

Age, particularly age of exposure [12], is expected to affect impacts of PTEs on members and the likelihood of developing Post-Traumatic Stress Symptoms (PTSS). Within younger populations, exposure to trauma may have a greater negative impact if left untreated or unattended [13]. PTE exposure is thought to increase with age [14], with a number of risk factors (e.g., family environmental stressors and chronic adversities) being associated with a higher risk to trauma, leading to the development of PTSS [13].

SLS are frequently exposed to PTEs which may differ from other first responders [5, 6]. For example, during the 2021/2022 season surf lifesavers and lifeguards performed over 8,000 rescues, 58,000 first aid treatments, and 1.6 million preventative actions [15]. Despite different exposures, it is likely that mental health outcomes are similar, namely PTSS, depression, and anxiety. All states and territories have peer support or similar member support services accessible for surf lifesavers and lifeguards. Whereby, the peer support groups facilitate an opportunity for debriefing following a PTE, and if required, referral to additional support services. To date, there has been no research exploring this link between PTE exposure and mental health outcomes in SLS, in particular adolescents, yet understanding the potential risk and protective factors in this group is vital due to member retention and improving mental health coping strategies when dealing with PTEs.

Rooke and de Terte [4] presented the only study to examine exposure to PTEs, PTSS, and post-traumatic growth (PTG) in New Zealand SLS (N = 181). The findings revealed a relationship between PTEs experienced outside of SLS work and PTSS. Younger members (17–19 year olds) showed greater risk of PTSD than older lifeguards (27 + year olds), which also related to greater potential for PTG for younger lifeguards [4]. Interestingly, women showed a negative relationship associated with PTSS in comparison to men [4].

Protective factors, such as self-efficacy (an individual’s belief in their capacity to act in the ways necessary to reach specific goals) may also influence the adverse effects of exposure to PTEs and PTSS. For example, an individual’s perception/beliefs about their capacity to manage a PTE has been related to PTSS, with higher self-efficacy scores representing a protective coping mechanism to PTEs [16, 17].

Social support has also been identified as a protective factor for several mental health conditions including PTSS [18, 19] in emergency services personnel. For example, a recent study revealed that high levels of received social support was associated with lower levels of PTSS [20]. Conversely, lower levels of social support were reported in experienced emergency service personnel with lower scores associated with higher levels of PTSS [21, 22]. Rooke and de Terte [4] did not report a significant correlation between PTSS and social support measures, but did find older SLS scored higher in self-efficacy compared to younger members.

Attitudes towards mental health is a predictor of action and has been shown to relate to an individual’s response to mental health [23, 24]. Attitudes towards mental health problems can be measured in the following facets: community and family attitudes towards mental health problems, stigma awareness via external shame and internal shame, family-reflected shame, and self-reflected shame [24].

Negative attitudes towards mental health problems have been associated with poor help-seeking behaviour within police [25, 26, 27], leading to affected personnel experiencing mental health conditions as well as being marginalised from their community [28]. In contrast, positive attitudes towards mental health problems have been promoted as a factor that may protect emergency personnel from experiencing mental health difficulties [29]. For example, positive attitudes towards mental health problems have been associated with better psychological well-being in military personnel [29]. While military roles may differ from that of SLS, they have the potential to experience similar exposure to PTEs [29].

**Fig. 1:**
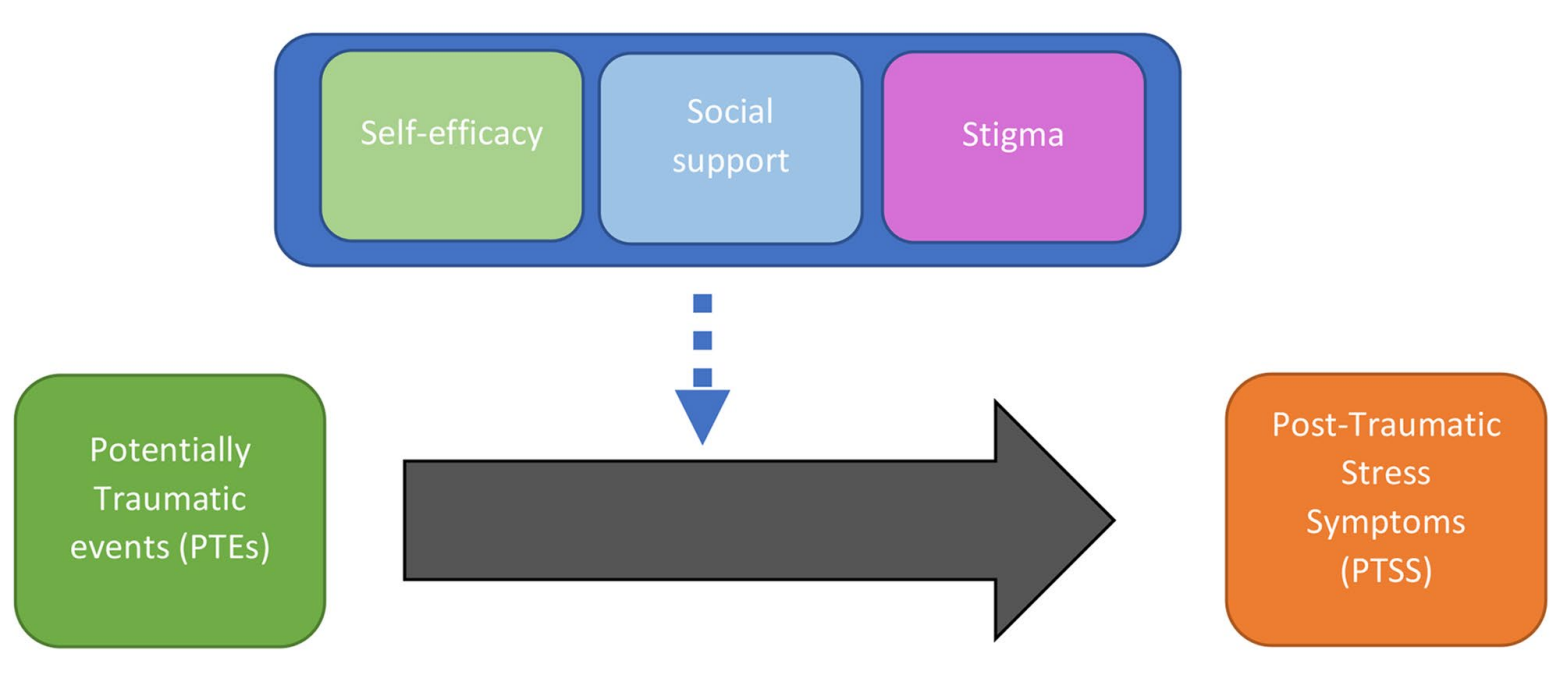
Framework for assessing the mental health of Australian lifesavers and lifeguards

To address the paucity of research on mental health of Australian surf lifesavers and lifeguards, and to extend the work of Rooke and de Terte [4], this paper presents a preliminary study that explores the mental health of Australian SLS members aged 13 years old and above. The risk factors that are referred to in this paper include gender, age, and years within SLS. The impact and influence of protective factors, including self-efficacy, social support, and attitudes towards mental health problems, are also explored. Specifically, we examine the relationship between PTEs and PTSS, self-efficacy, social support, and attitudes towards mental health problems in a preliminary study of adolescent and adult Australian surf lifesavers and lifeguards (refer to Fig. 1).

Based on existing research in other first responder personnel, we hypothesised there would be a positive relationship between PTEs and PTSS, and that PTSS would differ according to gender. We also hypothesised that self-efficacy, social support, and attitudes towards mental health problems would be protective against PTSS.

## Materials and methods

The preliminary survey was developed by a collaborative team of industry and academic professionals, then reviewed across the SLS movement to assess the appropriateness of the language and questions for intended participants and to ensure it addressed the intended purpose. The survey was structured with demographic questions, followed by several validated tools that assess exposure PTEs, PTSS, self-efficacy, social support, and attitudes towards mental health problems. Two separate anonymous, online surveys were developed: an adolescent survey for SLS members aged 13 to 17 years, and an adult survey for those aged 18 years and over. For context, in Australia, a person is generally considered an adult once they reach the age of 18 years in alignment with Australia’s eligibility to vote. Adult and adolescent versions only differed by items used to measure PTE exposure. Both surveys showed good internal consistency (Cronbach’s alphas ranged from 0.75 to 0.96; Table 1). The study was approved by the Human Research Ethics Committee (HREC 22265). All information collected during the surveys was confidential and responses were anonymous.

**Table 1:** Scales used and their internal consistency for the adult and adolescent survey

	Adult (Cronbach’s Alpha)	Adolescent (Cronbach’s Alpha)
**Life Events Checklist (LEC-5)**	Yes*	Partial*
**Adolescent Life Events (ALE)**	No	Yes*
**Posttraumatic Stress Disorder** **Checklist-5 (PCL-5)**	Yes (0.96)	Yes (0.93)
**Attitude Towards Mental Health** **Problems (ATMHP)**	Yes (0.87 to 0.96)	Yes (0.75 to 0.91)
**Social Support**	Yes (0.86)	Yes (0.86)
**Generalized Self-Efficacy Scale** **(GSE)**	Yes (0.91)	Yes (0.77)
**Experience of Mental Health**	Yes*	Yes*

*Since each item within the scale was measuring a different construct, analyses of internal consistency were inappropriate, and therefore, not run

### Participants

Participants were recruited through SLS membership database via email and specific SLS Facebook group pages. Data were collected online via the Qualtrics survey platform. The two surveys were distributed at different times (within 4 weeks) – the first to those eligible for the adults’ survey (18 + years old), the second to a smaller cohort of adolescent members (13–17 years old).

### Completion rates

The survey was sent out to 152 SLS members (82 adults, 70 adolescents) with 89 members (50 adults, 39 adolescents) responding to the online survey. For the purposes of this study, participants who had completed 100% of the survey were included in the analysis. In total, 57 SLS members responded to 100% of the survey (38 adults, 19 adolescents), with an overall completion rate of 64% (adult and adolescent). Of participants that did not complete the survey, 11 adults completed < 55% and 18 adolescents completed < 40%. To reduce burden, participants could save responses and return later. Non-patrolling members were removed from analyses (N = 4; n = 2 adolescents, and n = 2 adults) to focus on those who actively patrolled. For the purposes of this study, participants who had completed 100% of the survey were included in the analysis.

### Demographic measures

Demographic measures included gender, age, membership role in SLS, the state in which their club was located, years as a member of SLS, and years spent as an active patrolling member.

### Adults life events

The Life Events Checklist (LEC-5) screens for the occurrence of PTEs throughout an adult’s life [30]. The checklist included in the adult version contains 16 items depicting events likely to be traumatic. All 16 items were used in the adult survey, but only four items of the LEC-5 were included in the adolescent survey. A further nine items were included with the LEC-5 in the adult version relating to SLS [4]. Seven items were previously developed explicitly for SLS [4], and one item related to the COVID-19 pandemic followed by a final item used to screen for any other PTE not otherwise specified. The LEC-5 allowed participants to indicate how the PTE was experienced using the following multiple response options: “1 = Happened to me, 2 = Witnessed it, 3 = Learned about it, 4 = Within SLS, 5 = Outside of SLS, 6 = Not sure, 7 = Doesn’t apply, 8 = Experienced < 18 years old (not included in the adolescent survey), and 9 = Experienced 18 or older (not included in the adolescent survey)”.

Items were summed (except for “Not sure” and “Doesn’t apply” which were coded as zero) and trauma was categorized into four scores: global, direct, trauma within SLS, and trauma outside of SLS [4, 30]. A total of 25 items were included in the final adult modified LEC-5 (each scored to the value of one) with the scoring included in Table 2.

**Table 2:** Trauma domains

	Direct Trauma	Global trauma	Within SLS trauma	Outside of SLS trauma
**Definition**	Trauma experienced by the participant	Trauma the participant has either experienced, witnessed, or learnt about	Trauma that has occurred within SLS	Trauma that has occurred outside of SLS
**Answers participants needed to select within the survey to be categorized into each trauma domain**	“happened to me”	“happened to me, “witnessed it”, OR “learned about it”	“happened to me, OR witnessed it, AND “within SLS”	“happened to me, OR witnessed it, AND “outside of SLS”
**Adult survey score**	Between 0 and 25	Between 0 and 75	Between 0 to 50	Between 0 to 50
**Adolescent survey score**	Between 0 and 162	Between 0 and 54	Between 0 to 108	Between 0 to 108

SLS: Surf Life Saving

### Adolescent life events

The adolescent survey included the following items: 41-item Adolescent Life Events Stress Scale [31], plus four items from LEC-5 (which were deemed appropriate to include for the adolescent population), the seven SLS specific items [4], one item related to the COVID-19 pandemic (due to the situation and effect in Australia at the time of the survey being released), followed by a final item used to screen for any other PTE not otherwise specified. Response options were modified to be in line with the LEC-5, and was reduced to seven multiple response options “1 = Happened to me, 2 = Witnessed it, 3 = Learned about it, 4 = Within SLS, 5 = Outside of SLS, 6 = Not sure, 7 = Doesn’t apply”. Noting, “Experienced < 18 years old” and “Experienced 18 or older” was removed.

Items were summed (except for “Not sure” and “Doesn’t apply” which were coded as zero) and trauma was categorized into four scores: global, direct, trauma within SLS, and trauma outside of SLS [4, 30]. A total of 54-items were included (each scored to the value of one), with scoring included in Table 2.

### Post-traumatic stress

Both the adult and adolescent surveys included the 20-item Post-traumatic Stress Disorder Checklist-5 (PCL-5) to measure presence and severity of PTSS experienced within the past month [32], validated in adolescents as young as 12 years old (Liu et al., 2016). Each symptom was rated on a five-point scale ranging from “0 = Not at all, 1 = A little bit, 2 = Moderately, 3 = Quite a bit, 4 = Extremely”. Items are summed to provide a total severity score (0–80): higher scores indicated more severe PTSS. Scores of ≥ 33 indicated PTSS in both adults and adolescents [32]. However, a confirmed diagnosis of PTSD can only be made by a clinician [32].

### Self-efficacy

Both surveys included the Generalized Self-Efficacy Scale (GSE) which measures an individual’s belief in their capacity to act in the ways necessary to reach specific goals [33]. The scale comprises 10 items measured on a four-point scale ranging from “1 = Not at all, 2 = Hardly true, 3 = Moderately true, 4 = Exactly true”, and overall scores were calculated by summing the values of the 10 items. Higher scores represented higher perceived self-efficacy.

### Social support

A modified version of perceived social support was included in both surveys [34]. Modifications to reflect SLS context included rephrasing Items 1 and 3, and asking about support people more relevant within SLS context [4]. Item 1 changed from “How much does each of these people go out of their way to do things to make your work life easier for you” to “How much does each of these people go out of their way to do things to make your Surf Life Saving time more enjoyable for you?” and Item 3 “How much can each of these people be relied on when things get tough at work?” to “How much can each of these people be relied on when things get tough?”. Similarly, the original measure asks participants to report on “Your immediate supervisor”, “Other people at work”, and “Your wife, friends and relatives”, which had been altered to “A significant other (e.g., spouse/closest friend)”, “Family/friends”, “Surf Life Saving Peers”, “Patrol Captain”, and “Peer support worker”.

Participant social support responses for each item were on a five-point scale from “1 = Very little, 2 = A little, 3 = Some, 4 = A lot, 5 = A great deal” with the inclusion of a “Not applicable” option. Social support scores were calculated by averaging the scores, except items where participants selected “Not applicable”. Higher scores indicated greater social support available, provided by the support network.

### Attitudes towards mental health problems

For both surveys, the Attitude Towards Mental Health Problems scale (ATMHP; [24]) was used to explore the attitudes of SLS towards mental health problems. The scale comprises of 35 items measured on a four-point scale ranging from “1 = Do not agree at all, 2 = Agree a little, 3 = Mostly agree, 4 = Completely agree”. The scale includes five independent subscales measuring different facets of mental health attitudes (community and family attitudes towards mental health problems, stigma awareness through external and internal shame, reflected shame 1 (family-reflected) and reflected shame 2 (self-reflected)), each producing an individual score. Higher scores indicated negative attitudes towards mental health problems.

### Experience with mental health

Finally, participant mental health experience was explored using a scale created by the research team which focused on determining if the participant themselves, someone they grew up with, a friend, or a partner have experienced a mental health condition. Participants were able to indicate through a scoring each item the following: “1 = Yes, 2 = No, or 3 = Prefer not to say” for each item. Since each item was measuring a different construct, analyses of internal consistency were inappropriate.

### Statistical analyses

All analyses were performed using IBM SPSS 26. Internal consistency of the relevant scales was assessed using Cronbach’s alphas. Normality was assessed using the Shapiro-Wilk test and assumed where p > .05 (Supplementary Table 1). All *p*-values were 2-sided with demographic characteristics reported as M (*SD*) or N/% age. Binomial tests assessed whether participants had experience of mental health conditions in the combined sample. Chi-squared tests analysed differences in experience of mental health conditions across demographics.

To reduce the risk of a type I error, Holm’s stepwise adjustment for testing of multiple hypotheses was applied (Holm, 1979), with significance deemed at 0.05. Pearson’s correlations investigated relationships between PTEs, PTSS, self-efficacy, social support, attitudes towards mental health problems, age, years as a SLS member, and years patrolling. Spearman’s Rank was used for samples that violated the assumption of normality (Supplementary Table 1).

## Results

For an overall understanding of SLS members, data were pooled from both surveys where possible in the first instance, then analysed by age group (adult and adolescent), separately.

### Sample characteristics

Completed adolescent surveys (100%) took a minimum of 10 min, maximum 4.9 days, and on average 7.7 h. Completed adult surveys (100%) took a minimum of 6.15 min, maximum of 12 h, and on average 33 min. The high maximum and average survey completion time, particularly for adolescents, are a consequence of participants being able to save their answers and come back to complete the survey within a two-week time period.

Overall, participant mean age was 27.6 years (*SD* = 13.0), 52.6% identified as female (Table 3). Participants had patrolled on average 9.6 years (*SD* = 8.5), and had been a SLS member an average of 13.8 years (*SD* = 9.6). In the adult sample the average age was 34.1 years (*SD* = 12.1) with 55.3% females, and within the adolescent sample the average age was 15.4 years (*SD* = 1.2) with 47.4% females.

**Table 3:** Survey demographics for adolescent, adult, and combined sample

		Adolescents		Adults		Combined	
		n	%	n	%	N	%
**Gender**							
	Male	10	52.63	17	44.74	27	47.37
	Female	9	47.37	21	55.26	30	52.63
**Age Group**							
	13–17	19	100.00	0	0.00	19	33.33
	18–29	0	0.00	19	50.00	19	33.33
	30+	0	0.00	19	50.00	19	33.33
**State**							
	New South Wales	1	5.26	7	18.42	8	14.04
	Northern Territory	0	0.00	1	2.63	1	1.75
	Queensland	8	42.11	14	36.84	22	38.60
	South Australia	8	42.11	4	10.53	12	21.05
	Tasmania	2	10.53	4	10.53	6	10.53
	Victoria	0	0.00	5	13.16	5	8.77
	Western Australia	0	0.00	3	7.89	3	5.26
**Country of Birth**							
	Australia	78.95	15	34	89.47	49	85.96
	Other	21.05	4	4	10.53	8	14.04
**Roles**							
	Volunteer Surf Lifesaver	100.00	19	37	97.37	56	98.25
	Lifeguard	5.26	1	5	13.16	6	10.53
	Paid Employee	5.26	1	9	23.68	10	17.54
	Associate Member	0.00	0	1	2.63	1	1.75
	Multiple	10.53	2	11	28.95	13	22.81
**PTSS Criteria**							
	Met	5	26.3	6	15.8	11	19.3
	Not Met	14	73.7	32	84.2	46	80.7

PTSS: Post traumatic stress symptoms

Adult participants had, on average, been patrolling for an average of 13.0 years (*SD* = 8.6) and SLS members for 17.3 years (*SD* = 9.9). Adolescent participants had, on average, been patrolling for 2.9 years (*SD* = 1.2) and SLS members for 6.8 years (*SD* = 2.8).

The majority of participants were Australian-born (Adult: 89.5%, n = 34; Adolescent: 78.9%, n = 15, Table 3). In the adult sample, all SLS states/territories were represented with most participant clubs in Queensland (36.8%; n = 14) followed by New South Wales (18.4%; n = 7, Table 3). Not all SLS states/territories were represented by respondents in the adolescent version, with majority of participant clubs in Queensland (42.1%; n = 8), then South Australia (42.1%; n = 8, Table 3).

### Experience with mental health

Differences in mental health experience are shown in Table 4. Most participants did not experience any mental health conditions themselves (*p =* .049; Table 4), but had a friend who has a mental health condition (*p <* .001; Table 4). Adolescents were more likely to report growing up around someone with a mental health condition compared to adults (X^2^ = 8.564, *p =* .014; Table 4).

**Table 4:** Experience of mental health in the combined sample (adolescent and adult) sample

			Do you yourself suffer from any mental health conditions?			Did you grow up around someone who suffered from a mental health condition?			Do you have a friend that suffers from a mental health condition?			Are you currently in a relationship with someone who suffers from a mental health condition?		
			Yes	No		Yes	No		Yes	No		Yes	No	
		Total (%)	N (%)	N (%)	Test statistic (p-value)	N (%)	N (%)	Test statistic (p-value)	N (%)	N (%)	Test statistic (p-value)	N (%)	N (%)	Test statistic (p-value)
**Total responses**		57 (100)	18	33	(p =	29	28	(p =	42	14	(p <	4 (7)	53	(p < .001)
			(31.6)	(57.9)	0.049)	(50.9)	(49.1)	1.000)	(73.7)	(24.6)	0.001)		(93)	
**Sex**	Male	27	10	14	X2 1 =	13	14	X2 1 =	21	6	X2 1 =	3	24	X2 1 =
		(47.37)	(37)	(51.9)	0.806	(48.1)	(51.9)	0.153	(77.8)	(22.2)	0.215	(11.1)	(88.9)	1.317
	Female	30	8	19	(p =	16	14	(p =	21 (70)	8	(p =	1	29	(p = .336)
		(52.63)	(26.7)	(63.3)	0.396)	(53.3)	(46.7)	0.793)		(26.7)	0.761)	(3.3)	(96.7)	
**Age Group**	13–17	19	5	8	X2 2 =	14	5	X2 2 =	13	5	X2 2 =	1	18	X2 2 =
**(years)**		(33.33)	(26.3)	(42.1)	0.192	(73.7)	(26.3)	8.564	(68.4)	(26.3)	0.250	(5.3)	(94.7)	0.538
	18–29	19	6	13	(p =	10	9	(p =	15	4	(p =	1	18	(p = .764)
		(33.33)	(31.6)	(68.4)	0.909)	(52.6)	(47.4)	0.014)	(78.9)	(21.1)	0.883)	(5.3)	(94.7)	
	30+	19	7	12		5	14		14	5		2	17	
		(33.33)	(36.8)	(63.2)		(26.3)	(73.7)		(73.7)	(26.3)		(10.5)	(89.5)	
**Years patrolling**	0–9	31	10	15	X2 2 =	20	11	X2 2 =	23	7	X2 2 =	4	27	X2 2 =
		(54.39)	(32.3)	(48.4)	0.496	(64.5)	(35.5)	6.643	(74.2)	(22.6)	0.110	(12.9)	(87.1)	3.608
	10–19	19	6	13	(p =	8	11	(p =	14	5	(p =	0 (0)	19	(p = .165)
		(33.33)	(31.6)	(68.4)	0.780)	(42.1)	(57.9)	0.036)	(73.7)	(26.3)	0.947)		(100)	
	20+	7	2	5		1	6		5 (71.4)	2		0 (0)	7	
		(12.28)	(28.6)	(71.4)		(14.3)	(85.7)			(28.6)			(100)	
**Years as**	0–9	22	8	8	X2 2 =	16	6	X2 2 =	16	5	X2 2 =	3	19	X2 2 =
**member**		(38.6)	(36.4)	(36.4)	2.729	(72.7)	(27.3)	7.149	(72.7)	(22.7)	0.254	(13.6)	(86.4)	3.062
	10–19	21	7	14	(p =	7	14	(p =	15	6	(p =	0 (0)	21	(p = .216)
		(36.84)	(33.3)	(66.7)	0.255)	(33.3)	(66.7)	0.028)	(71.4)	(28.6)	0.881)		(100)	
	20+	14	3	11		6	8		11	3		1	13	
		(24.56)	(21.4)	(78.6)		(42.9)	(57.1)		(78.6)	(21.4)		(7.1)	(92.9)	

### Post-traumatic stress symptoms

For the combined sample, there was no difference in PTSS between genders, (t(55)=-0.899, *p* = .372). PTSS was not correlated with age (r_s_(55)=-0.185, *p* = .169), years as a SLSA member (r_s_(55)=-0.111, *p* = .411), or the number of years patrolling (r_s_(55)=-0.099, *p* = .466). Overall, 19.3% (total of 11 out of 57 participants) scored ≥ 33 on the PCL-5 (refer to Table 3 for adult and adolescent analysis). The mean adult PTSS score indicated a lower probability of PTSS symptoms, x̄=16.00, (Table 3). Concerningly, the mean score of PTSS in the adolescent sample was higher than the adult sample (x̄=23.74), but was still below the cut-off score of 33 that indicates probable PTSD symptoms (30, 35, 36) (refer to Table 3).

### Trauma

Correlations between PTEs and PTSS, split by age and gender are shown in Tables 4 and 5. Direct trauma (refer to Table 2) was positively correlated with PTSS in both adults, (*rs*(36) = 0.347, *p =* .033; Table 5) and adolescents, (*r*(17) = 0.480, *p =* .038; Table 5). No relationship between global trauma (refer to Table 2) and PTSS was observed in either the adult (*rs*(38) = 0.168, *p =* .314; Table 5) or adolescent sample (*r*(19) = 0.344, *p =* .150; Table 5).

**Table 5:** Correlations of PTSS split by age

			Global Trauma	Direct Trauma	Within SLS Trauma	Outside SLS Trauma
**Adolescent** **(13–17 years old)**	(n = 19)	Correlation	0.344	0.480*	.038^a^	.158^a^
		Sig. (2-tailed)	0.150	0.038	0.877	0.518
**Adult** **(18 + years old)**	(n = 38)	Correlation	.168^a^	.347^a^*	.308^a^	.178^a^
		Sig. (2-tailed)	0.314	0.033	0.060	0.284
	18–29 years old (n = 19)	Correlation	.134^a^	0.414	0.550*	.321^a^
		Sig. (2-tailed)	0.583	0.078	0.015	0.181
	30 + years old (n = 19)	Correlation	.101^a^	.125^a^	− .070^a^	.035^a^
		Sig. (2-tailed)	0.679	0.609	0.775	0.887

PTSS: Post traumatic stress symptoms; SLS: Surf Life Saving

* Correlation is significant at the 0.05 level (2-tailed) ^a^ Indicates a Spearman correlation was run for this variable, all other correlations were Pearson correlations

Adult males revealed positive correlations between PTSS and global trauma (*rs*(15) = 0.528, *p =* .029, Table 6) and PTSS and within SLS trauma (*rs*(15) = 0.507, *p =* .038, Table 6), while male adolescents showed positive correlations between PTSS and direct trauma (*r*(8) = 0.842, *p =* .002, Table 6). To further explore age as a risk factor, the adult sample was further divided into two age groups: those aged 18–29 years old and those aged 30 years and above. Younger adult (aged 18–29) PTSS was positively correlated with trauma experienced within SLS (*r*(17) = 0.550, *p =* .015; Table 5).

**Table 6:** Correlations of PTSS split by gender

			Global Trauma	Direct Trauma	Within SLS Trauma	Outside SLS Trauma
**Adolescents**	Male (n = 10)	Correlation	0.536	0.842**	− .110^a^	.226^a^
		Sig. (2-tailed)	0.110	0.002	0.762	0.530
	Female (n = 9)	Correlation	0.560	0.504	0.157	.079^a^
		Sig. (2-tailed)	0.117	0.167	0.687	0.841
**Adults**	Male (n = 17)	Correlation	.528^a^*	.390^a^	.507^a^*	.422^a^
		Sig. (2-tailed)	0.029	0.121	0.038	0.092
	Female (n = 21)	Correlation	− .091^a^	.366^a^	.173^a^	− .040^a^
		Sig. (2-tailed)	0.696	0.103	0.453	0.864

PTSS: Post traumatic stress symptoms; SLS: Surf Life Saving

* Correlation is significant at the 0.05 level (2-tailed)

** Correlation is significant at the 0.01 level (2-tailed)

^a^ Indicates a Spearman correlation was run for this variable, all other correlations were Pearson correlations

### Protective factors

Relationships between PTSS and self-efficacy and social support were also explored using correlations, split by age and gender (Tables 7 and 8).

**Table 7:** Correlations of PTSS split by age

			GSE score	Social support total	ATMHP1 attitudes towards mental health problems	ATMHP2 external shame	ATMHP3 internal shame	ATMHP4 reflected shame 1	ATMHP5 reflected shame 2
**Combined**		Correlation	− .171^a^	− .302^a^*	0.3.29^a*^	.444^a**^	.403^a^**	.586^a^**	.193^a^
		Sig. (2-tailed)	0.206	0.023	0.012	<0.001	0.002	< 0.001	0.151
		N	56	57	57	57	57	57	57
**Adolescent** **(13–17 years old)**		Correlation	0.541*	0.062	.339^a^	.316^a^	0.197	0.516*	.187^a^
		Sig. (2-tailed)	0.017	0.800	0.156	0.188	0.418	0.024	0.444
		N	19	19	19	19	19	19	19
**Adult** **(18 + years old)**		Correlation	− .440^a^**	− .482^a^**	.392^a^*	.589^a^**	.472^a^**	.566^a^**	.115^a^
		Sig. (2-tailed)	0.006	0.002	0.015	< 0.001	0.003	< 0.001	0.490
		N	37	38	38	38	38	38	38
	18–29 years old	Correlation	− 0.212	− 0.471*	.534^a^*	.633^a^**	0.593**	0.442	0.125
		Sig. (2-tailed)	0.399	0.042	0.019	0.004	0.007	0.058	0.611
		N	18	19	19	19	19	19	19
	30 + years old	Correlation	− .673^a^**	− .513^a^*	.237^a^	.509^a^*	.380^a^	.575^a^*	.038^a^
		Sig. (2-tailed)	0.002	0.025	0.329	0.026	0.109	0.010	0.878
		N	19	19	19	19	19	19	19

PTSS: Post traumatic stress symptoms; ATMPH: Attitudes Towards Mental Health

* Correlation is significant at the 0.05 level (2-tailed)

** Correlation is significant at the 0.01 level (2-tailed)

^a^ Indicates a Spearman correlation was run for this variable, all other correlations were Pearson correlations

**Table 8:** Correlations of PTSS split by gender

			GSE score	Social support total	ATMHP1 attitudes towards mental health problems	ATMHP2 external shame	ATMHP3 internal shame	ATMHP4 reflected shame 1	ATMHP5 reflected shame 2
**Adolescents**	Male	Correlation	0.623	0.136	0.527	.650^a^*	0.029	0.487	.198^a^
		Sig. (2-tailed)	0.054	0.709	0.118	0.042	0.936	0.153	0.583
		N	10	10	10	10	10	10	10
	Female	Correlation	0.733*	0.261	0.223	.154^a^	0.278	0.535	0.330
		Sig. (2-tailed)	0.025	0.497	0.564	0.693	0.468	0.138	0.387
		N	9	9	9	9	9	9	9
**Adults**	Male	Correlation	− .638^a^**	− .494^a^*	.000^a^	.567^a^*	.240^a^	.353^a^	.055^a^
		Sig. (2-tailed)	0.006	0.044	1.000	0.018	0.353	0.164	0.833
		N	17	17	17	17	17	17	17
	Female	Correlation	− .313^a^	− .444^a^*	.629^a^**	.554^a^**	.605^a^**	.662^a^**	.126^a^
		Sig. (2-tailed)	0.179	0.044	0.002	0.009	0.004	0.001	0.587
		N	20	21	21	21	21	21	21

PTSS: Post traumatic stress symptoms; ATMPH: Attitudes Towards Mental Health

* Correlation is significant at the 0.05 level (2-tailed)

** Correlation is significant at the 0.01 level (2-tailed)

^a^ Indicates a Spearman correlation was run for this variable, all other correlations were Pearson correlations

Self-efficacy was negatively correlated with PTSS for adults, (*rs*(35)=-0.440, *p* = 006; Table 7), and positively correlated with PTSS for adolescents, (r(17) = 0.541, *p* = .017; Table 7). Further gender analyses found a negative correlation between PTSS and self-efficacy in adult males (*rs*(15)=-0.638, *p* = .006; Table 8) and a positive correlation in adolescent females (r(7) = 0.733, *p* = .025; Table 8). PTSS was negatively correlated with self-efficacy for older adults (*rs*(17)=-0.673, *p* = .002; Table 7).

The combined sample (both adults and adolescents) showed a negative correlation between PTSS and social support (rs (55)=-0.302, *p* = .023; Table 7). Social support in adults was found to have a negative correlation with PTSS (*rs*(36)=-0.482, *p* = .002; Table 7), but not with adolescents (r(17) = 0.062, *p* = .800; Table 7). When dividing further by age, PTSS was negatively correlated with social support in both older adults (30 + years old; rs(17)=-0.513, p = .025; Table 7) and young adults (18–29 years old; r(17)=-0.471, *p* = .042; Table 7), as was PTSS and self-efficacy for older adults (rs(17)=-0.673, *p* = .002; Table 7). PTSS and social support was negatively correlated in both adult males (rs(15)=-0.494, *p* = .044; Table 8) and females (rs(19)=-0.444, *p* = .044; Table 8).

After Holm’s adjustment (Holm, 1979), the combined sample revealed positive correlations with PTSS and negative attitudes towards mental health problems (*rs* 55) = 0.329, *p* = .012; Table 7), external shame (*rs*(55) = 0.444, *p* < .001; Table 7), internal shame (*rs*(55) = 0.403, *p* = .002; Table 7), and reflected shame (*rs*(55) = 0.586, *p* < .001; Table 7). Most of the measures of attitudes towards mental health were positively correlated with PTSS for adults (i.e., negative attitudes towards mental health problems, (*rs*(36) = 0.392, *p* = .015), stigma awareness with external shame, (*rs*(36) = 0.589, *p* < .001), internal shame, (*rs*(36) = 0.472, *p* = .003), and reflected shame 1, (*rs*(36) = 0.566, *p* < .001), while no relationship was found in for the measure reflected shame 2 (*rs*(36) = 0.115, *p* = .490; Table 7). Conversely, no relationships were observed for these measures in the adolescent sample after Holm’s adjustment (Table 7). When dividing further by age, after Holm’s adjustment (Holm, 1979), PTSS was positively correlated with external shame (*rs*(17) = 0.633, *p* = .004; Table 7) and internal shame (r(17) = 0.593, *p* = .007; Table 7) in young adults (18–29 years old). After Holm’s adjustment (Holm, 1979) none of the analyses for older adults (30 + years old) were significant (Table 7).

When split by gender, only adult females showed a significant correlation between the measures of attitudes towards mental health and PTSS with attitudes towards mental health problems, *r*_*s*_(19) = 0.629, *p* = .002, external shame/ awareness, *r*_*s*_(19) = 0.554, *p =* .009, internal shame, *r*_*s*_(19) = 0.605, *p =* .004, and reflected shame 1 (family-reflected shame), *r*_*s*_(19) = 0.662, *p =* .001. While no relationship was found in for the measure reflected shame 2 (self-reflected shame), (*r*_*s*_(19) = 0.126, *p =* .587; Table 8).

## Discussion

This study is the first to investigate the relationship between trauma (PTEs) and PTSS, the risk factors of gender, age, and years of service, and the protective factors of self-efficacy, social support, and attitudes towards mental health problems within the Australian SLS context. A significant relationship between PTEs (global and in SLS role) and PTSS for adolescent was revealed, with adolescent males showing a significant relationship between PTEs and PTSS when compared with female adolescents. A significant relationship between higher self-efficacy and social support and lower levels of PTSS was detected in adult participants, but higher social support was related to increased PTSS for adolescents. Higher PTSS was surprisingly related to negative attitudes towards mental health problems in adult participants. Adolescents may experience additional stress when ‘re-living’ the PTE experience through discussing with social support networks or perhaps the belief is that they cannot cope with the environment/culture and that their friendship group will find out [37, 38]. In contrast, adults with greater life experience in dealing with PTE may become disconnected with the experience or are aware of the benefits of talking with social support networks [39–41]. However, given the small sample size, this interpretation must be taken with caution and highlights the need for further investigation. This is the first paper to explore mental health within SLS across a diverse scope of PTSS-related contexts unique to SLS patrolling roles, and to include adolescent members of SLS.

Overall, this study revealed a relationship between PTEs and PTSS in Australian surf lifesavers and lifeguards. This preliminary study is the first to characterise exposure across four types of PTEs: (i) global trauma, (ii) direct trauma, (iii) trauma within SLS, and (iv) trauma outside of SLS.

For adolescents a positive relationship between trauma and PTSS is proposed, however, this was not evident for adults. The latter contradicts Rooke and de Terte [4] where a positive relationship with PTEs and PTSS was observed. Rooke and de Terte [4] noted that younger participants (17–19 year olds) experienced higher PTSS compared to older participants (27 + year olds), similar to the current study where adolescents had higher PTSS than the adults. There is no comparative research for adolescents, however, it is plausible that adolescents who continue within SLS may be exposed to an accumulation of trauma and be at greater risk of developing PTSS. There is evidence in an Australian first responder population (over 21,000 police and emergency service workers) that those who had worked more than 10 years were almost twice as likely to experience psychological distress and six times more likely to experience symptoms of PTSD [5].

Notably, surf lifesavers patrol in team environment (minimum of 3 people in a team) whereby lifeguards patrol as a solo individual. For surf lifesavers, roles and tasks are allocated to all members by the leader of team (e.g., Patrol Captain) in preparation that a PTE may arise (e.g., swimmer with tube, board paddler, first aid, on lookout for ambulance arrival). Meanwhile, lifeguards are required to have the qualifications and ability to perform a patrol by themselves. Nonetheless, debriefs occur for both lifesavers and lifeguards who experience a PTE. Depending on the state/territory, further peer support or similar member support services are accessible for SLS members.

Adolescent males showed a positive relationship between direct trauma and PTSS, suggesting younger male SLS members (13–17 year olds) have more symptoms compared with older members (18 + year olds). Interestingly, PTSS is common in youth with approximately two-thirds of youth (aged 13–17 years old) exposed to PTEs [42]. However, with adolescent SLS little is known on how protective factors (self- efficacy, social support, and attitudes towards mental health problems) impact on PTE exposure compared to adolescents who are not first responders. This highlights opportunities for early intervention in education, awareness, and training being important for coping with PTEs. There were no other gender-related associations, which contrasts with previous research where females have reported higher PTSS compared to male counterparts [4, 43, 44].

Self-efficacy and social support, and their relationship with PTSS, were explored under the premise that the more social support an individual has and the greater the belief in self-ability to overcome certain situations, the less likely they would have PTSS. Self-efficacy was negatively correlated with PTSS for both adults and adolescents, suggesting that how SLS perceive certain situations, and how SLS behave in response to different situations (e.g., PTEs) can influence vulnerability to PTSS [46, 47]. This is consistent with some, but not all previous research as adolescents have been absent in Australian and international literature on this topic. For example, high levels of self-efficacy have been linked to lower PTSS in psychiatric and emergency nurses [47], yet higher self-efficacy was revealed as a risk factor for PTSS among emergency response personnel [47]. A potential disadvantage of higher self-efficacy may be drawn from the premise that emergency personnel may display overconfidence when exposed to PTEs and prefer to cope alone rather than within a team environment [47]. A key driver of self-efficacy as a protective factor is it impact on how we reflect on PTEs, e.g., individuals with higher self-efficacy scores are more likely to effectively recover after PTEs [46]. Self-efficacy is a critical coping component, with higher scores promoting PTSS recovery and resilience [47].

Social support was found to have a negative relationship with PTSS for the adult sample only. This is consistent with the large body of scientific literature in the first responder population that promotes social support as a protective factor against mental health difficulties such as PTSS [4, 45–47].

Finally, a positive relationship between PTSS and attitudes towards mental health problems revealed that as PTSS increased so did negative attitudes towards oneself and others’ perceptions of their mental health. Specifically, adults with higher PTSS were more likely to: perceive others to have negative perceptions of mental health problems, think others would look down on them if they had a mental health problem, report shame if they had a mental health problem, and think they brought shame on their families if they had a mental health problem. Interestingly, how others would perceive them if a family member had a mental health problem was not correlated with PTSS. This suggests negative attitudes towards mental health problems associated with having PTSS is introspectively targeted, which is a form of shame and can lead to long-term mental health difficulties. This exploratory study begins to address the knowledge gap in SLS and mental health, and confirms the need to further investigate mental health within SLS members.

### Limitations

A limitation of this study is that sample size comprises ~ 1% of SLS members. This study was intended as a preliminary, therefore findings may not be fully representative of SLS. The relatively small sample size may mean that our study is underpowered, and future studies should be conducted with larger samples to confirm our findings. Recruitment was also limited to direct email and specific SLS social media channels. Therefore, only regular email and social media users may have seen the survey invitation. Also, possible perceptions that the survey may contain sensitive topics, or a lack of interest in the topic from members, survey fatigue [48], and the anticipated time commitment may have adversely affected response rates. However, the overall 67% completion rate for this preliminary study suggests a readiness within the SLS community to engage in this type of research. The different life events checklist scales used in the adolescent and adult survey may also have impacted the completion rates. Both surveys were created to mitigate time burden versus risk to maintain the relevance to the target populations.

Further, this data was collected in 2020, during the impact of the COVID-19 pandemic where measures of distress, perception of stressful events, self-efficacy, and overall well-being may have been negatively affected during this time. There is also the likelihood that, for those experiencing psychological conditions, the topic of mental health may be confronting as we did not account for the age at first occurrence, severity, or chronicity of PTEs, all of which may impact mental health of an individual following exposure [49]. Consequently, potential self-selection bias of those individuals who completed the survey may have already been highly attuned to the distress within SLS roles and the impact of the pandemic [50]. These factors may have been barriers for survey completion (100%) with an overall (adolescent and adult survey) incomplete response rate of 36% and a decline rate found only in the adolescent sample of 13%. For those that did not complete the survey, 11 adult participants completed less than 55% (majority ceasing after the demographics and 2 participants ceasing after the LEC-5), while 18 adolescent participants completed less than 40% (ceasing after the demographic questions).

## Conclusions

This important preliminary study highlights the relationship of trauma-related experience with the psychological functioning of Australian SLS members. This research has not been previously conducted in the Australian Surf Life Saving context. The breadth of the age range, the adaptation of tools to SLS, and the use of targeted measures for first responders significantly extends emerging work in this area and provides a novel contribution to trauma-related research. Even though this study demonstrates the effective application of these measures, it only represents 1% of SLS, highlighting the need for a more extensive survey to gain a greater understanding of the well-being mental health within the Australian SLS movement. Understanding effects of exposure to PTEs during SLS duties on stress and related mental health outcomes are crucial to developing supportive programs for these forgotten first responders.

## Electronic supplementary material

Below is the link to the electronic supplementary material.

Supplementary Table 1. Shapiro Wilk p-values for not normally distributed data.

## Data Availability

The datasets generated during the current study are not publicly available due to consent not being provided for data sharing but are available from the corresponding author on reasonable request and with permission of CQUniversity and Surf Life Saving Australia.
