# Supplementary material for: A preliminary study investigating the neglected domain of mental health in Australian lifesavers and lifeguards
Source: BMC Public Health. 2023 May 31;23:1036. doi: 10.1186/s12889-023-15741-5 (PMC10231295; doi:10.1186/s12889-023-15741-5)
Supplement: Supplementary file 1 — Supplementary Table 1. Shapiro Wilk p-values for not normally distributed data. [file 12889_2023_15741_MOESM1_ESM.docx]

**Supplementary Table 1. Shapiro Wilk p-values for not normally distributed data.**

|  | | **Scale** | ***P*-value (*P*-value with missing data)** |
| --- | --- | --- | --- |
| **Combined** |  | PCL-5 | .003 (.005)^a^ |
| **Adults** |  | PCL-5 | .002 (.003)^a^ |
|  | Male | PCL-5 | .009 |
|  | Female | PCL-5 | .014 (.022)^a^ |
|  | 18-29 years old | Global trauma | .034 |
|  |  | Outside of SLS trauma | .046 |
|  |  | Attitudes towards mental health practices | .003 |
|  |  | External shame | .004 |
|  |  | Reflected shame 1 | .027 |
|  |  | Reflected shame 2 | < .001 |
|  | 30+ years old | PCL-5 | .003 |
| **Adolescent** |  | Attitudes towards mental health practices | .012 |
|  |  | External shame | < .001 |
|  |  | Reflected shame 2 | .004 |
|  |  | Within SLS trauma | .003 |
|  |  | Outside SLS trauma | < .001 |
|  | Males | Within SLS trauma | .002 |
|  |  | Outside SLS trauma | < .001 |
|  |  | External shame | .020 |
|  |  | Reflected shame 2 | .003 |
|  | Female | External shame | .012 |
|  |  | Outside SLS trauma | .005 |

**^a^ =** One of the participant’s was missing data for GSE but was still analysed in the data. So that is why two p-values are listed.
